# The Urine Output Response to Low-Dose Diuretic Challenge Predicts Tolerance to Negative Fluid Balance in Mechanically Ventilated, Critically Ill Patients

**DOI:** 10.7759/cureus.65824

**Published:** 2024-07-31

**Authors:** Jun Kataoka, Ryo Uchimido, Takushi Santanda, Tadanori Nabeshima, Yoshihisa Fujimoto, Yasuhiro Norisue, Shigeki Fujitani

**Affiliations:** 1 Department of Pulmonary and Critical Care Medicine, Nerima Hikarigaoka Hospital, Tokyo, JPN; 2 Department of Intensive Care Medicine, Tokyo Medical Dental University, Tokyo, JPN; 3 Department of Pulmonary and Critical Care Medicine, Tokyo Bay Urayasu Ichikawa Medical Center, Urayasu, JPN; 4 Department of Emergency and Critical Care Medicine, St. Marianna University School of Medicine, Kawasaki, JPN

**Keywords:** : acute kidney injury, critical illness, fluid balance, acute respiratory failure, diuretics

## Abstract

Background and objective

Although early diuretic use and negative fluid balance (NFB) have been associated with lower mortality in mechanically ventilated patients, some patients are not tolerant to NFB. Little is known about whether urine output response after the diuretic administration predicts NFB tolerance in mechanically ventilated patients. Hence, we conducted this study to look into this.

Methods

This was a single-center, prospective, observational study. We included mechanically ventilated patients who were hemodynamically stable with bilateral pulmonary opacities on chest radiography and planned to be diuresed per our fluid removal protocol. In the protocol, a low dose of furosemide adjusted to each patient’s estimated glomerular filtration rate (eGFR) was administered, and then we started to measure urine outputs hourly for four hours. Tolerance to NFB was defined as “absence of hypotension, fluid resuscitation and vasopressors use, and acute kidney injury during fluid removal”. We investigated whether the urine output predicts the tolerance to NFB during fluid removal treatment.

Results

A total of 60 mechanically ventilated patients were included. Notably, 80% (48/60) of the patients were tolerant to NFB. All hourly and cumulative urine output measurements during the first four hours after the first diuretic administration were significantly higher in the NFB-tolerant group than in the non-tolerant group. Among all hourly and cumulative urine output measurements, the first four-hour cumulative urine output showed the highest area under the receiver operating characteristic curve (AUC) of 0.83 for predicting the tolerance to NFB. Multivariate logistic regression analysis adjusted for the urine output two hours before the diuretic use showed that each 100-mL increase in the first four-hour cumulative urine output was significantly associated with an increased odds ratio (OR) of the tolerance to NFB [adjusted odds ratio (aOR): 1.53; 95% CI: 1.11-2.15].

Conclusions

Based on our findings, the first four-hour cumulative urine output after the first low dose of diuretic administration might help predict tolerance to NFB during fluid removal treatment in mechanically ventilated, critically ill patients.

## Introduction

Recent research has shown that fluid overload is associated with poor outcomes in critically ill patients [1-5]. In hemodynamically stable patients with acute respiratory distress syndrome (ARDS), a conservative fluid management strategy including diuretic administration can improve lung function and shorten the duration of mechanical ventilation [6]. Although early diuretic use and negative fluid balance (NFB) are linked to lower mortality in critically ill patients [7,8], premature initiation of diuresis can increase the chances of non-pulmonary organ failure, such as acute kidney injury (AKI) [9].

It is difficult to predict tolerance to NFB before starting fluid removal; hence, the timing of initiating diuresis varies widely among clinicians. One study has demonstrated that a high mitral inflow E-wave to early diastolic mitral annulus velocity ratio (E/E’ ratio) measured by echocardiography predicts tolerance to NFB well [10]. Another study has stated that a positive passive leg raising (PLR) test before fluid removal predicts the occurrence of subsequent hypotension during renal replacement therapy (RRT) in critically ill patients [11].

Furosemide is commonly used in critically ill patients with a positive fluid balance [12]. In the renal tubular lumen, furosemide inhibits the luminal Na-K-Cl cotransporter in the thick ascending limb of the loop of Henle, thereby causing natriuresis and increased urine flow [13,14]. The urine output response to furosemide might be a tool to assess renal tubular function and has been shown to predict progression to AKI and the need for RRT [15-17]. Our ICU has used a protocol to record the urine response to a small dose of furosemide before aiming for NFB as a finding possibly useful for assessing the volume status based on the hypothesis that a response to the first low dose of diuretic use could identify patients with adequate cardiac output and renal perfusion, and, hence, may identify patients with tolerance to NFB. However, no studies have yet evaluated this hypothesis and the validity of the urine output response to diuretics as a tool to predict tolerance to NFB, which may prevent adverse events of forcing a negative net fluid balance with an increased dose of diuretics or other means. In this study, we aimed to investigate whether the urine output after the first diuretic use predicts tolerance to NFB in mechanically ventilated, critically ill patients.

## Materials and methods

Study design

**Figure 1:**
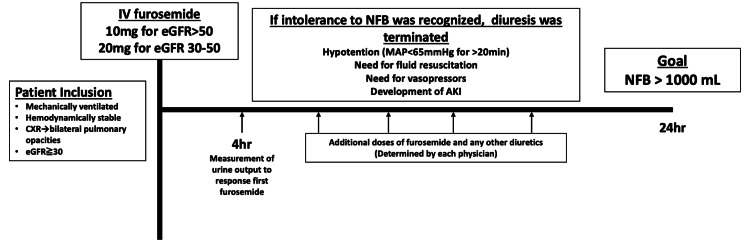
Fluid removal protocol AKI: acute kidney injury; CXR: chest X-ray; eGFR: estimated glomerular filtration rate; MAP: mean arterial pressure; NFB: negative fluid balance

This was a single-center, prospective, observational study. We developed a fluid removal protocol for mechanically ventilated patients before starting fluid removal, and this protocol has been regularly used for critically ill patients on mechanical ventilation. Patients were excluded from the fluid removal protocol if the primary reason for requiring mechanical ventilation was heart failure, or if the volume status of patients was not deemed to have reached the stabilization phase [18]. The study was conducted in the medical-surgical ICU from October 2016 to August 2018, using a pre-established fixed fluid removal protocol (Figure 1) that had been implemented since April 2016.

Fluid removal protocol

In our fluid removal protocol (Figure 1), mechanically ventilated patients (18 years or older) who were hemodynamically stable with bilateral pulmonary opacities on chest radiography and for whom diuresis was planned were included. Hemodynamic stability was defined as a mean arterial pressure (MAP) >65 mmHg in the absence of any vasopressors and no evidence of volume depletion (volume status in the stabilization phase or de-escalation phase). Patients who met any of the following criteria were excluded: (1) baseline estimated glomerular filtration rate (eGFR) <30 ml/minute/1.73 m^2^; (2) Kidney Disease Improving Global Outcome (KDIGO) AKI stage 3; (3) cause of intubation was congestive heart failure and cardiac surgery; (4) prior use of diuretics during the ICU stay; (5) prior introduction of RRT during the ICU stay; (6) no Foley catheter at the time of inclusion; (7) evidence of obstructive uropathy; (8) allergy to loop diuretics; (9) evidence of active bleeding; and (10) known pregnancy.

A low dose of furosemide was administered (10 mg for eGFR >50 ml/minute/1.73 m^2^, 20 mg for eGFR 30-50 ml/minute/1.73 m^2^) [19] to patients included in our fluid removal protocol. Urine outputs were then measured every hour, and cumulative urine outputs for four hours were calculated [19]. After the measurement, the patients were diuresed to achieve an NFB of more than 1000 mL in 24 hours with additional doses of furosemide and any other diuretics, as needed. Additional doses of furosemide and any other diuretics were determined at the discretion of each physician. As soon as intolerance to NFB was recognized, diuresis was terminated. Intolerance to NFB was defined as the development of hypotension (defined as MAP <60 mmHg for >20 minutes or a decrease >40 mmHg), the need for fluid expansion (500 mL of crystalloid or colloid solution or blood products in 30 minutes), the need for vasopressors, or the development of AKI within 24 hours after starting diuresis (defined as development or progression by the KDIGO definition) [20].

Statistical analysis

After inclusion, the following clinical and laboratory data were recorded: baseline characteristics at ICU admission [age, sex, BMI, comorbidities, Acute Physiology and Chronic Health Evaluation (APACHE) Ⅱ score, cause of intubation, PaO_2_/FiO_2_ ratio], and parameters at inclusion (cumulative fluid balance after ICU admission, MAP, heart rate, ventilator settings, PaO_2_/FiO_2_ ratio, serum creatinine, serum albumin, serum lactate, urine output during two hours before furosemide use). Before administering the diuretic, transthoracic echocardiography (TTE) was performed to obtain several parameters, such as respiratory variations of inferior vena cava (IVC) diameter, mitral inflow E-wave velocity (E) and A-wave velocity (A), early diastolic lateral mitral annulus velocity (E’) measured by tissue Doppler imaging, left ventricular ejection fraction (LVEF), and LV outflow tract velocity time integral (LVOT-VTI).

Firstly, patients’ baseline characteristics and characteristics at the time of administration of the first diuretic, including vital signs, blood test results, and the TTE parameters were summarized in both the NFB-tolerant group and the non-tolerant group. Then, summary statistics, medians with interquartile range (IQR) for quantitative variables, and proportions for the qualitative variables were used to compare the two groups. Then, urine outputs per hour and cumulative urine outputs were compared between the two groups and plotted on a box plot.

Second, the association of these urine output measurements with tolerance to NFB was further investigated by plotting receiver operating characteristic (ROC) curves to compare their ability to predict tolerance to NFB. The area under the ROC curves (AUC) was calculated, and then the Youden index was used to determine the optimal cut-off point for sensitivity, specificity, and positive and negative predictive values. The sample size was estimated by considering the incidence of tolerance to NFB as 90%, with an AUC of 0.8, a type 1 error of 0.05, and a power of 80% [10]. The sample size was estimated to be 60 patients.

Third, multivariable logistic regression analysis was performed to estimate the odds ratio (OR) for tolerance to NFB for every 100-ml increase in the four-hour cumulative urine output that showed the highest AUC of all urine output measurements. The model was adjusted for urine output two hours before the diuretic use that was significantly different between the tolerant and non-tolerant groups. The Hosmer-Lemeshow goodness-of-fit test was used to assess the goodness of the fitted model.

The Mann-Whitney U test was used to compare quantitative variables. Fisher’s exact test was used to compare qualitative variables. For measures of association, 95% CIs were computed, and significance was defined as a two-tailed p-value of less than 0.05. Because this study was exploratory, there was no correction for multiple testing. All statistical analyses were performed with the IBM SPSS Statistics version 22.0 (IBM, Corp., Armonk, NY) and R version 4.0.2 package (R Foundation, Vienna, Austria).

Ethical approval and consent to participate

This study was approved by the Institutional Review Board at Tokyo Bay Urayasu Ichikawa Medical Center (TBUIMC) (No. 213) and was registered with UMIN-CTR Clinical Trial (UMIN000027134) on 26/04/2017. A waiver of informed consent was obtained because the study was part of routine practice in our hospital. Opt-out information was published at the following URL: https://tokyobay-mc.jp/opt_out.

## Results

Baseline characteristics

**Figure 2:**
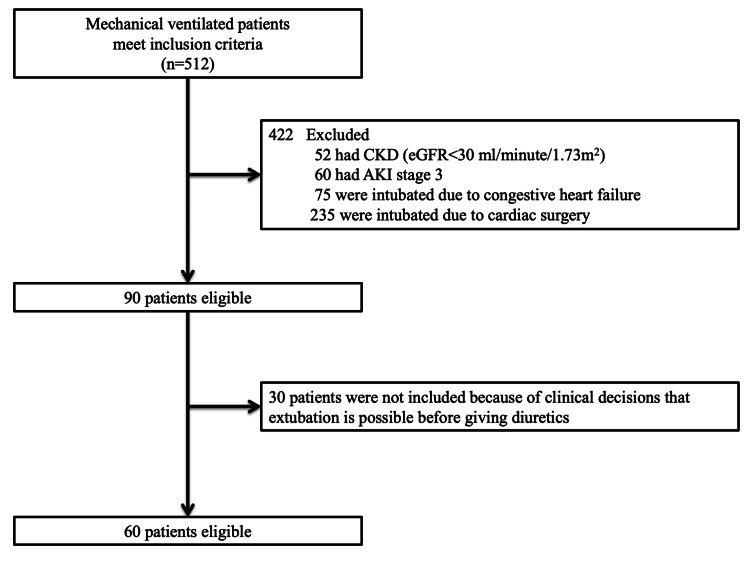
Flowchart depicting patient selection AKI: acute kidney injury, CKD: chronic kidney disease, eGFR: estimated glomerular filtration rate

A total of 60 mechanically ventilated patients were included (Figure 2). Of note, 68% of patients (41/60) had septic shock, and 50% (30/60) had moderate to severe ARDS; 80% (48/60) were tolerant to NFB (Table 1). Of the 12 patients not tolerant to NFB, seven developed hypotension, and five developed AKI due to the presence of oliguria; six were given 10 mg, and the remaining patients were given 20 mg of intravenous furosemide as the initial dose. The rate of patients who received 10 mg and 20 mg of furosemide did not differ significantly between the two groups (p=0.52, data not shown). No significant differences in age, sex, BMI, comorbidities, baseline serum creatinine, or the APACHE Ⅱ score were observed between the two groups of patients at baseline. The fluid balance at 24 hours after administration of the first diuretic was significantly less in the NFB group [-1495 (-1952 - -1277) mL vs. 370 (15-827) mL, p<0.01]. The duration from first extubation after inclusion in the fluid removal protocol tended to be shorter in the NFB group [2 (1-4) days vs. 3 (2-6) days, p=0.07]. There was no significant difference in in-hospital mortality between the two groups (27% vs. 33%, p=0.67).

**Table 1:** Baseline characteristics of patients in both groups APACHE: Acute Physiology and Chronic Health Evaluation; ARDS: acute respiratory distress syndrome; BMI: body mass index; IQR: interquartile range

Variable	Tolerance to NFB (n=48)	Poor tolerance to NFB (n=12)	P-value
Age, years, median (IQR)	76 (66-83)	66 (56-79)	0.20
Male sex, n (%)	29 (60)	5 (42)	0.25
BMI, kg/m^2^, median (IQR)	22.7 (18.6-25.7)	19.3 (15.8-22.1)	0.10
Comorbidities, n (%)			
Hypertension	24 (50)	7 (58)	0.61
Diabetes mellitus	9 (18)	4 (33)	0.28
Baseline creatinine, mg/dl, median (IQR)	0.74 (0.62-0.97)	0.61 (0.47-0.86)	0.08
APACHE 2 score, median (IQR)	19 (15-21)	21 (16-25)	0.35
Cause of intubation, n (%)			
Pneumonia	21 (44)	9 (75)	0.10
Extrapulmonary sepsis	23 (48)	2 (17)	0.06
Other	4 (8)	0	
Postoperative patients, n (%)	18 (37)	2 (16)	0.18
Septic shock, n (%)	34 (70)	7 (58)	0.41
PaO_2_/FiO_2_ ratio on admission, median (IQR)	203 (144-277)	178 (97-266)	0.37
Moderate to severe ARDS, n (%)	23 (48)	7 (58)	0.52

Characteristics at the time of administration of the first diuretic

Table 2 shows the characteristics at the time of administration of the first diuretic in patients with and without tolerance to NFB. No significant difference was observed between the two groups in MAP, heart rate, PaO_2_/FiO_2_ ratio, serum albumin, or serum lactate. Cumulative fluid balance from ICU admission to inclusion was comparable between the two groups [5484 (3464-8192) mL in patients with tolerance to NFB vs. 4695 (1267-6053) mL in patients with intolerance to NFB, p=0.16]. The urine output during the two hours before the first diuretic use was significantly higher in patients with tolerance to NFB, compared to patients with non-tolerance to NFB [100 (70-140) mL vs. 50 (30-90) mL p=0.01]. All TTE measurements, including the maximum IVC diameter, the variation of IVC collapsibility, LVEF, LVOT-VTI, E/A, and E/E’, were not significantly different between the two groups.

**Table 2:** Characteristics at the time of administration of the first diuretic AKI: acute kidney injury; E/A: E-wave velocity/A-wave velocity ratio; E/E’: E-wave velocity/early diastolic lateral mitral annulus velocity ratio; HR: heart rate; ICU: intensive care unit; IQR: interquartile range; IVC: inferior vena cava; LVEF: left ventricular ejection fraction; LVOT-VTI: left ventricular outflow tract velocity time integral; MAP: mean arterial pressure; TTE: transthoracic echocardiography

Variable	Tolerance to NFB (n=48)	Poor tolerance to NFB (n=12)	P-value
Days in the ICU before inclusion, days, median (IQR)	3 (2-4)	3 (2-4)	0.74
Fluid balance from admission, ml, median (IQR)	5484 (3464-8192)	4695 (1267-6053)	0.16
MAP, mmHg, median (IQR)	80 (74-89)	83 (71-95)	0.90
HR, /min, median (IQR)	77 (68-89)	84 (71-98)	0.21
Lactate, mg/dl, median (IQR)	10.9 (8.4-14.8)	14.6 (9.2-17.8)	0.12
PaO_2_/FiO_2_ ratio, median (IQR)	288 (218-345)	253 (187-321)	0.51
Creatinine, mg/dl, median (IQR)	0.84 (0.63-1.22)	0.70 (0.54-1.00)	0.23
AKI, n (%)	32 (66)	6 (50)	0.29
Albumin, mg/dl, median (IQR)	2.3 (1.9-2.7)	2.2 (1.8-2.9)	0.70
Urine output before two hours, ml, median (IQR)	100 (70-140)	50 (30-90)	0.01
ml/kg, median (IQR)	1.6 (1.2-2.2)	1.1 (0.7-1.8)	0.04
TTE measurements, median (IQR)			
Maximum IVC diameter, mm	21 (17-23)	22 (21-23)	0.42
IVC collapsibility, %	20 (10-32)	10 (7-20)	0.08
LVEF, %	55 (50-60)	58 (40-60)	0.76
LVOT-VTI, cm	17.7 (15.8-20.1)	15.8 (13.5-24.7)	0.84
Cardiac output, L	3.9 (2.8-5.4)	4.4 (3.7-5.4)	0.45
E/A	0.94 (0.88-1.15)	0.78 (0.69-1.01)	0.08
E/E’	9.2 (7.3-12.6)	6.6 (4.4-13.6)	0.24

Urine output responses after the first diuretic administration in the NFB-tolerant group vs. non-tolerant group

**Figure 3:**
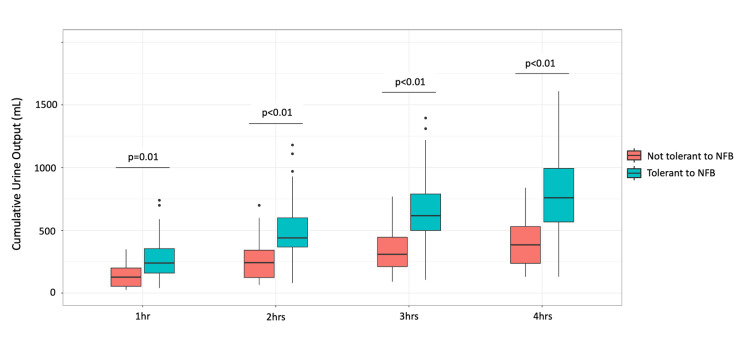
Boxplots showing cumulative urine output per hour after diuretic administration NFB: negative fluid balance

**Figure 4:**
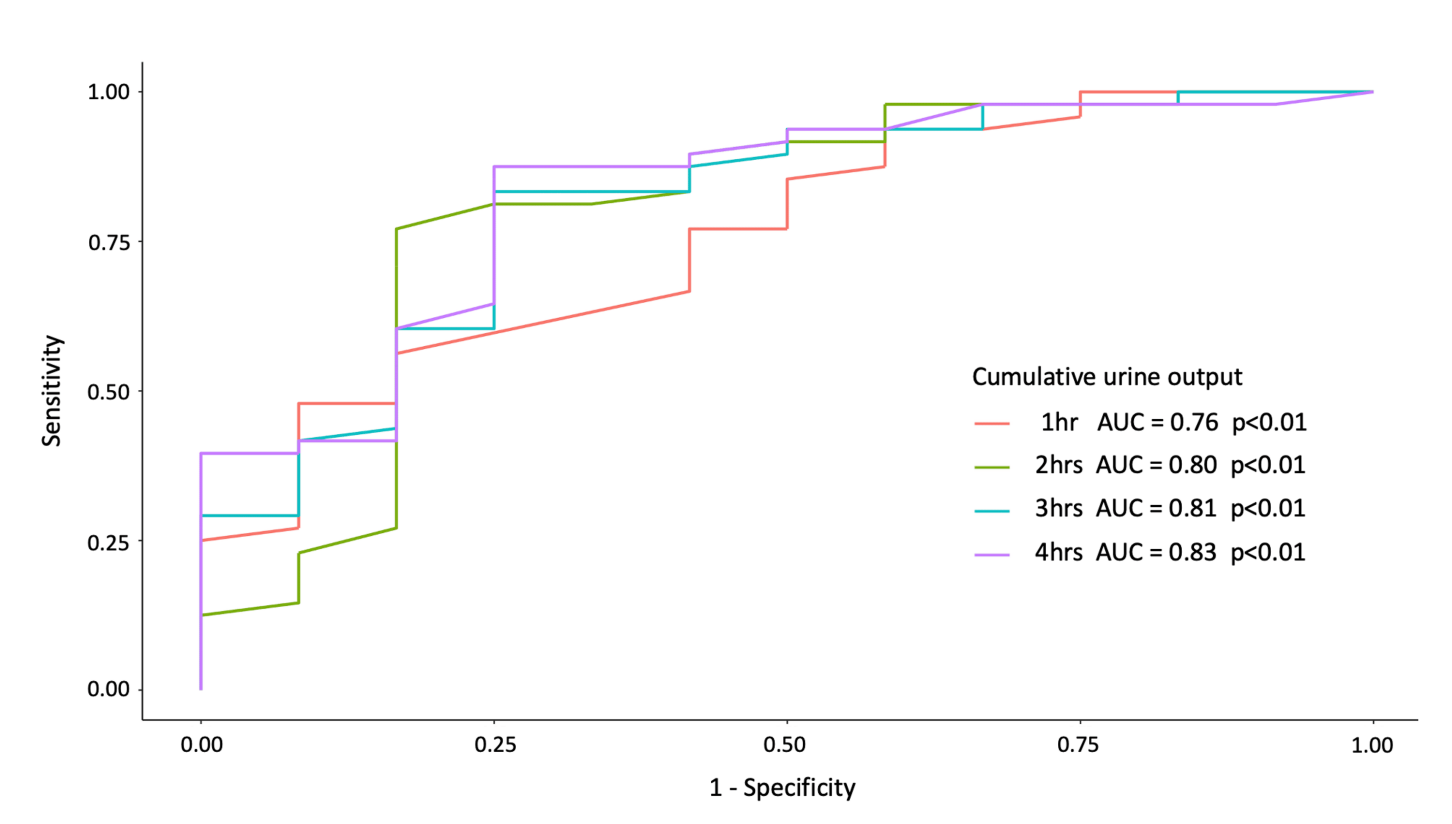
ROC curves for cumulative urine output four hours after diuretic administration (1 hour, 2 hours, 3 hours, 4 hours) The areas under the curves are 0.76, 0.80, 0.81, and 0.83, respectively AUC: area under the receiver-operator characteristic curve; ROC: receiver operating characteristic curve

All hourly and cumulative urine output measurements during the first four hours after the first diuretic administration were significantly higher in the NFB-tolerant group than in the non-tolerant group (Table 3, Figure 3). The first four-hour cumulative urine output after the first diuretic administration had the highest AUC to predict tolerance to NFB (AUC: 0.83, 95% CI: 0.69-0.96, p<0.01) (Table 3, Figure 4). In the overall sample, the cutoff point for urine output of 500 ml during the four hours after the first diuretic use showed a sensitivity of 0.85, a specificity of 0.75, a positive predictive value of 0.56, and a negative predictive value of 0.93.

**Table 3:** Urine output following the first diuretic AUC: area under the receiver operating characteristic curve; IQR: interquartile range; NFB: negative fluid balance

Variable	Tolerance to NFB (n=48)	Poor tolerance to NFB (n=12)	P-value	AUC
The urine output of each hour, ml, median (IQR)				
0-1 hour	240 (160-370)	130 (40-200)	0.01	0.76
1-2 hours	200 (140-280)	100 (40-150)	0.01	0.75
2-3 hours	140 (90-250)	70 (40-90)	<0.01	0.81
3-4 hours	130 (70-200)	50 (30-70)	<0.01	0.80
Accumulation of the urine output, ml, median (IQR)				
2 hours	440 (370-600)	240 (120-340)	<0.01	0.80
3 hours	620 (500-790)	310 (210-450)	<0.01	0.81
4 hours	760 (560-1000)	380 (220-580)	<0.01	0.83

Multivariate logistic regression analysis adjusted for the urine output during the two hours before the diuretic use showed that each 100-mL increase in the first four-hour cumulative urine output was significantly associated with an increased OR for tolerance to NFB [adjusted odds ratio (aOR): 1.53; 95% CI: 1.11-2.15]. The Hosmer-Lemeshow goodness-of-fit test showed good calibration of the fitted model (p=0.31).

## Discussion

Our findings showed that the urine output after the first diuretic use predicted tolerance to NFB with good sensitivity and a good negative predictive value in mechanically ventilated patients undergoing diuresis for clinical evaluation of hypervolemia. This result could be explained by our hypothesis that the response to the low dose of diuretic administration may reflect adequate cardiac output and renal perfusion, leading to tolerance to NFB. Since furosemide was used as a diuretic in this study, another possible pathophysiology other than renal perfusion to explain the results is the interaction between furosemide and serum albumin. Furosemide is tightly bound to serum albumin and is actively secreted into the tubular lumen in the convoluted tubule [21]), and a low serum albumin level may attenuate the effect of furosemide. Patients already in recovery may have higher serum albumin levels than those who are still critically ill. However, the serum albumin level was not significantly different between patients with tolerance and those with intolerance to NFB in this study.

In daily practice, it is often difficult to determine the appropriate timing to initiate fluid removal, and the time varies widely among physicians; thus, feasible and simple ways to predict tolerance to NFB are warranted. Although a previous study demonstrated that a high E/E’ ratio on echocardiography predicted tolerance to NFB in ARDS patients [10], and the E/E’ ratio on echocardiography had a better correlation with left ventricular filling pressure [22,23], all TTE measurements in the present study including E/E’ did not differ significantly between the groups. Another study showed that a positive PLR test before fluid removal predicted subsequent hypotension during renal replacement therapy in critically ill patients [11]. However, for the PLR test, cardiac output must be measured using a special device or echocardiography [24].

Thus, the present method might be simpler and more practical for predicting tolerance to NFB. This method may prevent the adverse effects of forced diuresis to achieve NFB with progressively increased doses of diuretics or other means such as ultrafiltration. This method may also lead to safe and earlier initiation of diuresis to achieve NFB, possibly followed by earlier extubation. It is important to note that patients with congestive heart failure for whom forced diuresis with increased doses of diuretics is often helpful for cardiac unloading and consequent improvement of organ perfusion were excluded.

The present study has some limitations. Firstly, this was a single-center study with a relatively small sample size, which warrants external validation of this method in future studies. Second, because patients with a baseline eGFR <30 ml/minute/1.73 m^2^ and with KDIGO AKI stage 3 criteria were excluded, it is unclear if the present method can be useful in predicting tolerance to NFB in such patients. Lastly, urine output before the first diuretic use also predicted tolerance to NFB, and further studies with larger sample sizes are needed to compare the AUCs of urine output before and after diuretic use.

## Conclusions

This study's findings showed that the first four-hour cumulative urine output after the first low dose of diuretic administration might help predict tolerance to NFB during fluid removal treatment in mechanically ventilated, critically ill patients. Additional interventional trials with larger sample sizes are warranted to confirm this finding.

## References

[1] Acheampong A, Vincent JL (2015). A positive fluid balance is an independent prognostic factor in patients with sepsis. Crit Care.

[2] Brotfain E, Koyfman L, Toledano R (2016). Positive fluid balance as a major predictor of clinical outcome of patients with sepsis/septic shock after ICU discharge. Am J Emerg Med.

[3] Payen D, de Pont AC, Sakr Y, Spies C, Reinhart K, Vincent JL (2008). A positive fluid balance is associated with a worse outcome in patients with acute renal failure. Crit Care.

[4] Messmer AS, Zingg C, Müller M, Gerber JL, Schefold JC, Pfortmueller CA (2020). Fluid overload and mortality in adult critical care patients-a systematic review and meta-analysis of observational studies. Crit Care Med.

[5] Claure-Del Granado R, Mehta RL (2016). Fluid overload in the ICU: evaluation and management. BMC Nephrol.

[6] Wiedemann HP, Wheeler AP, Bernard GR (2006). Comparison of two fluid-management strategies in acute lung injury. N Engl J Med.

[7] Shen Y, Zhang W, Shen Y (2019). Early diuretic use and mortality in critically ill patients with vasopressor support: a propensity score-matching analysis. Crit Care.

[8] Alsous F, Khamiees M, DeGirolamo A, Amoateng-Adjepong Y, Manthous CA (2000). Negative fluid balance predicts survival in patients with septic shock: a retrospective pilot study. Chest.

[9] McCoy IE, Montez-Rath ME, Chertow GM, Chang TI (2019). Estimated effects of early diuretic use in critical illness. Crit Care Explor.

[10] Allyn J, Allou N, Dib M (2013). Echocardiography to predict tolerance to negative fluid balance in acute respiratory distress syndrome/acute lung injury. J Crit Care.

[11] Monnet X, Cipriani F, Camous L (2016). The passive leg raising test to guide fluid removal in critically ill patients. Ann Intensive Care.

[12] Jones SL, Martensson J, Glassford NJ, Eastwood GM, Bellomo R (2015). Loop diuretic therapy in the critically ill: a survey. Crit Care Resusc.

[13] Burg M, Stoner L, Cardinal J, Green N (1973). Furosemide effect on isolated perfused tubules. Am J Physiol.

[14] Dirks JH, Seely JF (1970). Effect of saline infusions and furosemide on the dog distal nephron. Am J Physiol.

[15] Chawla LS, Davison DL, Brasha-Mitchell E (2013). Development and standardization of a furosemide stress test to predict the severity of acute kidney injury. Crit Care.

[16] Lumlertgul N, Peerapornratana S, Trakarnvanich T (2018). Early versus standard initiation of renal replacement therapy in furosemide stress test non-responsive acute kidney injury patients (the FST trial). Crit Care.

[17] Chen JJ, Chang CH, Huang YT, Kuo G (2020). Furosemide stress test as a predictive marker of acute kidney injury progression or renal replacement therapy: a systemic review and meta-analysis. Crit Care.

[18] De Backer D, Cecconi M, Chew MS (2022). A plea for personalization of the hemodynamic management of septic shock. Crit Care.

[19] Oh SW, Han SY (2015). Loop diuretics in clinical practice. Electrolyte Blood Press.

[20] Khwaja A (2012). KDIGO clinical practice guidelines for acute kidney injury. Nephron Clin Pract.

[21] Bowman RH (1975). Renal secretion of [35-S]furosemide and depression by albumin binding. Am J Physiol.

[22] Dokainish H, Zoghbi WA, Lakkis NM, Al-Bakshy F, Dhir M, Quinones MA, Nagueh SF (2004). Optimal noninvasive assessment of left ventricular filling pressures: a comparison of tissue Doppler echocardiography and B-type natriuretic peptide in patients with pulmonary artery catheters. Circulation.

[23] Combes A, Arnoult F, Trouillet JL (2004). Tissue Doppler imaging estimation of pulmonary artery occlusion pressure in ICU patients. Intensive Care Med.

[24] Cherpanath TG, Hirsch A, Geerts BF, Lagrand WK, Leeflang MM, Schultz MJ, Groeneveld AB (2016). Predicting fluid responsiveness by passive leg raising: a systematic review and meta-analysis of 23 clinical trials. Crit Care Med.

